# Understanding Experiences of Fibromyalgia Patients Involved in the Fimouv Study During COVID-19 Lockdown

**DOI:** 10.3389/fpsyg.2021.645092

**Published:** 2021-07-20

**Authors:** Claire Colas, Audrey Jumel, Marie-Pierre Vericel, Nathalie Barth, Jessica Manzanares, Julie Goutte, Luc Fontana, Léonard Féasson, David Hupin, Jessica Guyot

**Affiliations:** ^1^Univ. Lyon, UJM-Saint-Etienne Autonomic Nervous System Research Laboratory, SAINBIOSE INSERM, U1059, Saint-Etienne, France; ^2^Department of Clinical and Exercise Physiology, University Hospital Center, Saint-Etienne, France; ^3^Univ. Lyon, UJM-Saint-Etienne Chaire Santé des Ainés - Ingénierie de la Prévention, Saint-Etienne, France; ^4^Univ. Lyon, UJM-Saint-Etienne Chaire ActiFS, Saint-Etienne, France; ^5^Gerontopole AURA, Saint-Etienne, France; ^6^Pain Center, University Hospital Center, Saint-Etienne, France; ^7^Department of Internal Medicine, University Hospital Center, Saint-Etienne, France; ^8^Department of Occupational and Environmental Medicine, University Hospital Center, Saint-Etienne, France; ^9^Univ. Lyon, Univ. Lyon 1, Univ. St Etienne, Univ. Gustave Eiffel, IFSTTAR, UMRESTTE, UMR_T9405, Saint-Etienne, France; ^10^Univ. Lyon, UJM-Saint-Etienne Interuniversity Laboratory of Human Movement Biology, EA 7424, Saint-Etienne, France; ^11^Department of Medicine, K2, Solna, Karolinska Institutet, Stockholm, Sweden

**Keywords:** COVID-19, lockdown, fibromyalgia, physical activity, social rhythms, quality of life, qualitative study

## Abstract

**Introduction:** The COVID-19 pandemic implied a period of lockdown for the general population, increasing the risk to develop some physical or mental disorders. In fibromyalgia patients, these disorders are part of the large clinical picture of the syndrome. Fibromyalgia management is especially based on a regular practice of physical activity. Lockdown imposed a break in rhythms, requiring a restructuring of scheduling. Thus, the present study aimed to investigate the experiences of fibromyalgia patients during COVID-19 lockdown using a qualitative analysis.

**Method:** 19 patients (52 ± 9 years old) who completed a 3-month therapeutic education and/or supervised physical activity program were invited to participate (Fimouv study, Trial registration: ClinicalTrials.gov NCT04107948). A sociologist collected data by means of semi-structured interviews and analyzed them using thematic content analysis.

**Results:** Lockdown exacerbated the main symptoms of fibromyalgia, but adjusting the rhythms of life to fluctuations of these symptoms allowed a better quality of life. Patients felt the lack of physical activity and 68% found alternatives to remain physically active. The reduction of social constraints allowed them to better contend with their pathology. Fibromyalgia stopped being a main priority.

**Conclusion:** Lockdown was positively experienced by fibromyalgia patients. They linked the absence of physical activity with increased pain and fatigue. Nevertheless, reducing social constraints could be a key for fibromyalgia management, where symptoms seemed to take less space in everyday life.

**Clinical Trial Registration:** www.ClinicalTrials.gov, identifier: NCT04107948.

## Introduction

Like most countries in the world, France has been affected by the COVID-19 pandemic (or severe acute respiratory syndrome coronavirus 2, SARS-CoV-2) declared by the World Health Organization since March 11, 2020 (WHO, 2020). In November 2020, given the alarming figures of the second epidemic wave of SARS-CoV-2, the French government announced a new lockdown for a period of at least 1 month. This lockdown situation was experienced for the first time from March to May 2020 and disrupted everyone's lifestyle: non-essential businesses and structures were closed; and travel was allowed only for work, food, care, and individual physical activity within 1 km from home (Decree n ° 2020-260, 2020).

If a lockdown can reduce the virus contamination rate and limit the hospital overcrowding, it is not devoid of consequences. Several authors underlined deleterious effects on mental health, e.g., emotional disturbances, stress, anxiety, exhaustion, and depression (Brooks et al., 2020). In a qualitative analysis, people expressed a depressed mood during the period of isolation and sometimes felt the need for professional mental health support after only 2 weeks of lockdown (Williams et al., 2020). Lockdown affects the usual sleep rhythm for more than a third of adults who had not been infected with the virus (Wang et al., 2020; Zhao et al., 2021), characterized by more time spent in bed but with a lower quality of sleep (Cellini et al., 2020). All the studies have been unanimous about the significant impact of lockdown, with an increased risk for women, people who lack family support, and those who have a poor quality of sleep, psychological history and/or fear of getting infected with the virus (Jeong et al., 2016; Tang et al., 2020; Wang et al., 2020).

Most of these risk factors are found in fibromyalgia patients, predominantly females (Vincent et al., 2013). Indeed, clinical manifestations of this syndrome are mainly characterized by the triad of pain, fatigue and sleep disturbances, affecting quality of life (Abeles et al., 2007; Häuser et al., 2010). The syndrome is the result of the interplay between many predisposing, triggering, and sustaining factors that fuel the vicious cycles of fibromyalgia. Thus, the term fibromyalgia syndrome is used as a common final pathway of multiple somatic, psychological and social contributions, specific to each patient (Ferrari, 2000; Littlejohn and Walker, 2002; Turk and Adams, 2016). Among the recurrent psychosocial manifestations of this syndrome, studies found mental disorders in nearly 97% of fibromyalgia patients (Miki et al., 2018), anxiety and depressive disorders (13–64% and 20–80%, respectively, Fietta et al., 2007), often supported by previous experiences of burnout, trauma and/or difficult life stories. In addition, sleep disorders were present in 90% of cases (Osorio et al., 2006). These sleep difficulties caused increased fatigue and modified painful perceptions by altering descending pain-inhibition pathways (Choy, 2015). These constraints led to a reduction in physical, professional and social activities and disrupted the different temporalities of life. In sociology, we differentiate four types of temporalities in daily life: biological time; economic time (working time); familial and cultural time; and personal time (Chesneaux, 1997). With chronic disease, these times become upsetting, imposing significant coping skills for patients (Grimaldi, 2006). Generally, this situation promotes social isolation and gradually leads to a decline in physical and functional capacities, deconditioning and fatigability, promoted by pain, fatigue, fear of movement and feeling of incapacity (Nijs et al., 2013; Turk and Adams, 2016). Patients gradually switch to physical inactivity and an increase in sedentary behavior in daily life.

In the absence of a curative drug treatment, scientists promote physical activity in all its forms in order to relieve symptoms (Figueroa et al., 2008; Häuser et al., 2010; Busch et al., 2011; Menzies et al., 2014; Bidonde et al., 2019). Actually, for this syndrome, adapted physical activity is recommended as a first-line treatment, as it is the only “strong for” therapy-based recommendation in the EULAR guidelines (Macfarlane et al., 2017). With lockdown, engaging and/or maintaining a regular practice of an adapted physical activity is difficult because of the closure of structures and trips restrictions limited to 1 km around the home, reducing the possibilities to exercise and promoting social isolation (Goethals et al., 2020).

Thus, during lockdown we observe a break in rhythms that is imposed not only on the practice of physical activity, but also on all spheres of life (work, family, leisure, etc.), requiring a restructuring of scheduling. Fibromyalgia patients are not spared from the multiple consequences of lockdown, which will add to the existing symptoms (Mohabbat and Mohabbat NML, 2020). Given the pre-existing weaknesses in terms of mental health and sleep, which are already very serious symptoms in fibromyalgia, and knowing that these patients accumulate several risk factors for developing symptoms related to lockdown (sex, sleep, psychological history, etc.), the fibromyalgia population could be particularly affected by the lockdown constraints (Batres-Marroquín et al., 2020; Cavalli et al., 2021). The purpose of the present study was to investigate the impact of COVID-19 lockdown on fibromyalgia patients in order to understand their experiences given the changes in everyday life and temporalities.

## Methods

**Figure 1:**
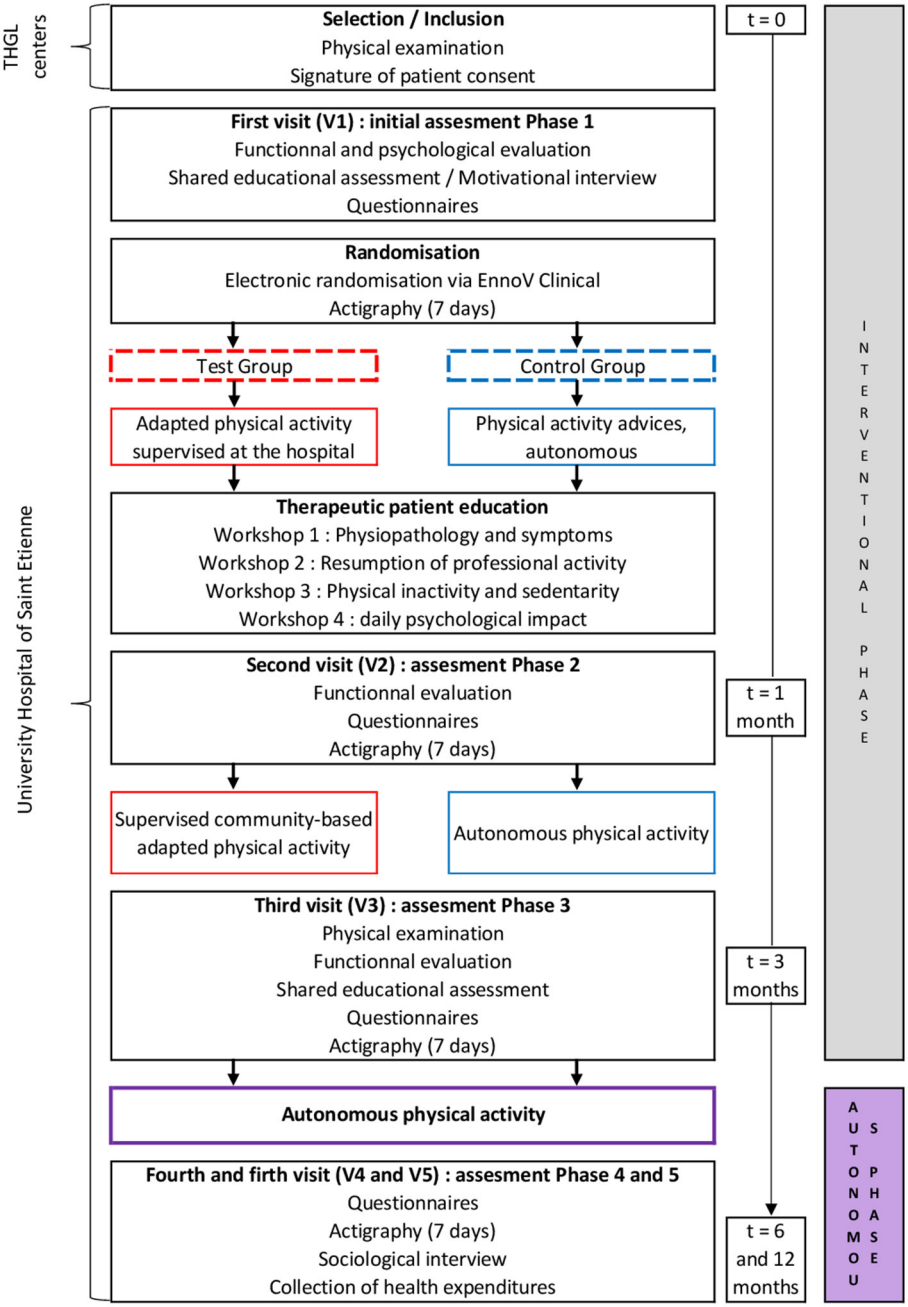
Study design of Fimouv. THGL, Territorial Hospital Group of Loire.

We conducted a qualitative survey in the research protocol entitled From Intent to Move [Fimouv (Colas et al., 2021); Trial registration: ClinicalTrials.gov NCT04107948; funded by the General Directorate of Healthcare, Health French Ministry, France]. This study aimed to optimize the care pathways of patients suffering from fibromyalgia by offering a supervised adapted physical activity program, added to a therapeutic patient education program. During the education sessions, study members (doctors, nurse, teacher in adapted physical activities, psychologist) raise patients' awareness of the benefits of physical activity with the aim of empowering them in their practice. The study team followed the patients for 1 year (Figure 1).

### Participants Selection

To be eligible for this qualitative study, the Fimouv participants (1) were over 18 years, (2) met the 2016 ACR fibromyalgia diagnostic criteria, (3) had completed the interventional phase of the Fimouv study (first 3 months, Figure 1), and (4) were volunteers. All the participants were contacted by e-mail to create a voluntary sample for telephone interview. Sociologists conducted the interviews for 1 month, that is from the 4th to the 8th week of first lockdown in France, allowing us to appreciate the changes in the responder's experiences of lockdown regarding the situation. All participants provided informed consent prior to participation according to the ethics committee (IRBN522020/CHUSTE), which approved the study.

### Data collection

To explore the objectives of the study, we developed a semistructured interview guide (Supplementary Material) divided into six main themes: an introductory part, followed by a feedback on the experience of lockdown with fibromyalgia, the place of COVID-19 in daily life, the link with family and social surroundings, the place of adapted physical activity during lockdown, and finally post-lockdown projection. A team member (AJ) tested this guide with a person with fibromyalgia before starting data collection. In all 19 people were questioned (16 women and three men), reflecting the figures highlighted in the literature regarding female predominance in fibromyalgia syndrome. Our respondents were between 38 and 70 years old, with an average age of 52 ± 9 years. The duration of the interviews ranged from 21 to 45 min. Their professional situation and living conditions during lockdown are presented in Table 1. Interviews were recorded and anonymized, transcribed verbatim and reviewed for accuracy by the interviewer. A full transcript will be followed by a thematic and comparative analysis of the content of the cross-data.

**Table 1:** Characteristics of interviewees.

**Anonymization**	**Date of interview (dd/mm/yy)**	**Duration of interview**	**Age**	**Living environment**	**Employment**
Laura	07/04/20	00:37:14	42	Apartment without exterior	Job search
Gabrielle	08/04/20	00:42:28	52	Detached house with a large plot	Partial unemployment
Bernard	08/04/20	00:33:04	53	House in the middle of the fields	Active
Vanessa	09/04/20	00:28:24	55	House with a small garden	Disability
Hillary	10/04/20	00:36:08	38	House with a garden	Disability
Corinne	14/04/20	00:27:38	58	House with a garden	Disability
Fabienne	15/04/20	00:38:04	52	Apartment with a terrace	Sick leave
Karine	15/04/20	00:45:13	44	Apartment without exterior	Teleworking
Blanche	17/04/20	00:31:58	43	Detached house with a garden	Disability
Tatiana	17/04/20	00:22:20	40	House with a garden	Partial unemployment
Florence	22/04/20	00:29:04	68	House with a small garden	Retired
François	24/04/20	00:37:15	58	House with a garden	Sick leave
Romane	28/04/20	00:29:22	56	Apartment with a balcony	Disability
Grace	28/04/20	00:27:04	63	House with a garden	Retired
Sophie	28/04/20	00:37:34	44	House with a garden	Active
Patricia	29/04/20	00:24:19	70	Apartment with a balcony	Retired
Perrine	04/05/20	00:21:04	59	House with a small garden	Teleworking
Marie	06/05/20	00:23:12	50	Apartment with a balcony	Disability
Florian	07/05/20	00:45:11	46	Duplex apartment with a small garden	Active

### Data Analysis

The inductive method allows, from raw data of patients' speeches, to report on experiences and key events in order to “concretely address the topic of interest and let the facts suggest the important variables” (Beaugrand, 1988). This method allowed us to constantly go back and forth between the field and the theory, considering changes in the health context in our case. To analyze our contents, we used the thematic content analysis of Bardin which is organized into three distinct stages: first the pre-analysis to formulate areas of research to create an analysis grid, then the exploitation of material by encoding data, and finally the processing of the results obtained in order to synthesize data (Bardin, 2013).

In an empirical or qualitative approach, adequate sample size is the one that reaches theoretical saturation (Mason, 2010). The latter is reached when the sociologist is unable to find additional information to improve the theory. Griffin and Hauser demonstrated that the amount of additional information decreased with the number of people interviewed according to a beta-binomial distribution (Griffin and Hauser, 1993). In this study, recruitment of participants was stopped once the thematic saturation was reached at 19 interviews.

## Results

### An Unprecedented Situation Which Disrupts the Daily Life of Fibromyalgia Sufferers

For the first time in France, the government implements a home-based lockdown for the general population. For individuals suffering from fibromyalgia, everyday life was completely upset with the restrictions for leaving home.

#### Disruption of Normal Patterns of Life and Stress

“*The announcement of the lockdown was atrocious […] only anxiety attacks and stress” (Laura, 42 years old)*. The lifestyle disorganization linked to the lockdown favored the exposure to stress for 11 of 19 interviewees with multiple factors, sometimes common with the general population: “*I am more stressed, we think about everything but I think it's the same for everyone” (Karine, 44 years old)*; and sometimes specific to their pathology: “*I am also worried about my medical follow-up.” (Hillary, 38 years old)*.

Firstly, as a general population, for people having a job, the economic crisis linked to the closure of businesses disrupted pace and conditions of work for an indefinite period. This home-based work situation caused weariness and fatigue among our respondents: “*I have more work to do, I don't feel like I am resting, […] that my days all look the same and that I can't do anything” (Karine, 44 years old)*. Lockdown delayed the job search and job interviews of our participants, causing them to feel stressed: “*I had a lot of job interviews before the lockdown, it all blew up so that's why I think I got really stressed out” (Laura, 42 years old)*.

Then as a patient, access to medical and paramedical care was limited or non-existent and could reinforce the feeling of exclusion related to the non-recognition and ignorance of fibromyalgia syndrome. Even if no drug treatment has proven its effectiveness to treat fibromyalgia, the chronic and fluctuating nature of this syndrome implied for most patients (more than 60% in our study) the use of some antalgics, antidepressants or antiepileptics continuously or occasionally depending on the intensity of the symptoms. With the first home lockdown, the access to the pharmacy was regulated and patients with chronic diseases (fibromyalgia or others) had seen their processing times and administration of care slowed down. However, it seemed that it did not affect the medication intake by fibromyalgia patients, which was sensibly the same than before the lockdown: “*No, there is no problem, I called my pharmacy who prepared everything […] and it was my husband who just took the bag from the pharmacy” (Blanche, 43 years old)*. Five other people expressed the absence of difficulties in accessing treatment. Generally, fibromyalgia patients require a medical follow-up by several professionals of hospitals. During the lockdown period, consultations were suspended, at best delayed, to make way for medical emergencies linked to COVID-19. Community-based doctors also were impacted since a lot of health professionals were requisitioned to respond to the health emergency. Medical and paramedical offices were closed or limited their activity, making the follow-up care more difficult in particular for sessions with physiotherapists: “*It's hard not to have physiotherapy, since the offices are closed we cannot follow the sessions” (Romane, 56 years old)*.

This exceptional situation provoked by COVID-19 grabbed everyone's attention and specially media interest. News channels and newspapers covered the events provoked by this health crisis and disclosed continuously potential anxiety-inducing information: “*I tell myself ‘now I avoid watching the news’, it is no use listening to every day, it stresses even more” (Gabrielle*, 52 *years old)*. Stress and fear of virus were all the more expressed if the close surrounding was affected, as in 37% of our sample: “*I have a neighbor who died and my nephew who had it [COVID-19; …], it took him several months to recover […] and I realized that it was dangerous, you have to be really careful” (Romane, 56 years old)*. Our respondents then showed a feeling of vulnerability toward this crisis, which pushed for the strictest respect of the lockdown: “*When it happened to our family I started to get scared and stressed and said you have to be careful so my doctor put me off work so I could stay at home” (François, 58 years old)*.

#### Exacerbation of Symptoms During Lockdown

Fibromyalgia clinical manifestations are mainly represented by the triad of pain, fatigue and sleep disorders. These three symptoms were all the more intensified by home lockdown: “*Fatigue, pain and lack of sleep, these are things that I have constantly at the moment” (Karine, 44 years old)*.

During “normal” periods (out of lockdown), sleep disorders are ubiquitous in the daily life of fibromyalgia sufferers: “*Sleep has always been my real problem. I don't sleep very well it's not just during lockdown” (François, 58 years old)*. They observed poor quality of sleep with nights that can be shortened, painful and restless: “*I can't fall asleep before 1:30am in the morning […] in the morning we put an alarm clock anyway and it's super hard” (Laura, 42 years old)*. Lockdown caused additional difficulties to fall asleep and therefore shorter nights and complicated awakenings. Also, one in two respondents noticed the regular occurrence of nightmares: “*No I don't sleep well at all, I often have nightmares and very agitated dreams” (Fabienne*, 52 *years old)*.

In fibromyalgia, pain is diffused in the whole body and is responsible for muscle stiffness: “*Pain point of view though, I am in pain all the time. It can be in the neck […], in the back, at the top of the back, the hips, well pain everywhere.” (Hillary, 38 years old)*. These are disabling musculoskeletal pains affecting mainly the upper back, neck, shoulders, arms, hands or hips. “*I was in so much pain […] I even had to take my pills to fall back to sleep at night” (Blanche, 43 years old)*. With lockdown, it seemed that the increase in pain had a negative impact on sleep, further accentuating the disorders observed.

Fatigue is a symptom frequently observed in fibromyalgia patients participating in the Fimouv program, showing periodic oscillations typical of fibromyalgia: “*It's true that since lockdown I still feel more tired” (Grace, 63 years old)*. Even if this state of fatigue fluctuates every day, 75% of people interviewed had noticed an increase during lockdown: “*I'm so painful and super tired from doing nothing” (Blanche, 43 years old)*.

### Finally, a Situation Suitable to a Life Rhythm for Oneself

Over weeks, everyone acclimates to the situation, new habits are put in place and stress decreases. We could have thought that people with fibromyalgia would feel more vulnerable with regard to the virus, particularly due to their pathology, but ultimately it is a feeling of security that predominates in their words: “*No, I'm not afraid and I tell myself that at home if I respect the government directives I don't have risks” (Gabrielle, 52 years old); “I don't leave home so I'm safe” (Blanche, 43 years old)*. If stress was present firstly, it gave way to more quietness from the second week of lockdown. Indeed, people found in this period an opportunity to set up new activities based on their own rhythm: “*I take the time to rest but I continue to be active” (François, 58 years old)*.

#### A Redefinition of Social Times

First of all, lockdown implied a reorganization of time. The usually predominant place of working time in daily life were reduced or temporarily stopped. Even if the professional activity continues, travels by car and/or by public transportation were greatly reduced, and these trips were very costly in energy for these people. Trips constraints for children tended to decrease, even if school continued at home: “*I do remote schooling for my two children, so it's a new activity that requires a lot of work and takes up a lot of my afternoons” (Tatiana, 40 years old)*. As much as possible, respondents organized themselves to reduce access to stores for food purchases, where there were sometimes long waiting lines that are unpleasant for people with fibromyalgia: “*I go shopping much less often and I realize that it is good and as soon as possible I have my fruits and vegetables delivered” (Bernard, 53 years old)*. This allowed people to have more space for familial and personal times: “*With my daughters, we cooked a lot” (Marie, 50 years old)*; “*I do a lot of drawings and paintings and there I got in a lot; whereas I never have the time to do it” (Laura, 42 years old)*.

The period was still conducive to pacing activities, switching between rest and adjusting task/occupation: “*I do work at my own pace because I get tired very quickly” (François, 58 years old)*. As a result of fatigue and with the altered sleep patterns during lockdown, patients needed to take care of themselves and take regular rest: “*I don't hesitate to lie down, take a nap when I feel like I need it” (Corinne, 58 years old)*. The period of lockdown was profitable and allowed one to have more time for oneself and a much less restrictive rhythm of life: “*I take time to rest, to sit on my terrace, it feels good I have fewer constraints” (Gabrielle, 52 years old)*. For 75% of interviewees, this time was used for listening to one's body, allowing one to live in harmony with fibromyalgia and adapt daily tasks to fluctuations in its symptoms: “*I do my activities at my own pace, I manage my schedule, I don't worry if I haven't finished what I had to do today I finish it the next day” (Corinne, 58 years old)*.

#### The Place of Physical Activity in Everyday Life: Feeling of Lack of Practice

“*I do much less physical activity […] with home-based lockdown” (François, 58 years old)*. The implementation of lockdown created restrictions on movement and consequently reduced the time devoted to physical activities. Interestingly, patients made the link between an increased fatigue and the inactivity imposed by lockdown: “*I notice that I miss my physiotherapy sessions twice a week a lot, as well as yoga, and it affects my fatigue and pain” (Blanche, 43 years old)*. Our patients, previously aware about physical activity with Fimouv, really realized the benefit of physical activity for their pathology. The absence or reduction of the practice allowed them to understand that adapted physical activity is a real way to fight symptoms. For them, this decrease in physical activity had repercussions on their physical function: “*I'm less well […] I really stiffen up as I do less activities” (Romane, 56 years old)*.

Thus, people with fibromyalgia were strongly advised to remain physically active to manage the multiple symptoms. To overcome this lack and the impossibility of practicing outside the home, patients organized new physical activity strategies. Also, 70% of people questioned succeeded to implement alternatives to remain physically active: “*I do a mix of stretching and exercises from my physiotherapist” (Florence, 68 years old)*; “*I'm trying to do yoga moves I found videos on Youtube” (Laura, 42 years old)*. In the long term, Fimouv's objective is to empower patients in their physical practice. Thus, in this unprecedented period, the resources provided by the program enabled two thirds of the respondents to set up their own physical activities. Being deprived of physical activity made them want to get involved.

#### An Opportunity to Re-establish Social Ties

“*What's really hard socially is that when you explain that you can't do something and in front of you we don't understand why, well we end up not wanting to go out with them anymore.” (Laura, 42 years old)*. Usually fibromyalgia patients tend to isolate themselves socially due to a lack of understanding of their pathology. With home lockdown, this social exclusion could have been accentuated: “*I know that I isolate myself even more, with what is going on I don't want to go out and meet people” (Vanessa, 55 years old)*. On the contrary, it appears that they lacked social ties, mainly those with loved ones: “*I miss seeing people with lockdown, it's not easy because a social link is important” (Fabienne, 52 years old)*. To handle it, new technologies have taken their place in homes: “*I have more communications with my brother, while before we called each other very, very little; and he even did video Zoom meetings. It's pretty funny and it allows us to see each other even from a distance.” (Laura, 42 years old)*. This period allowed them to reconnect with family members. The links within the home were also strengthened by an increase in family time in 63% of respondents: “*We do more things together with my children, more activities, it's important at this time” (Sophie, 44 years old)*. The care, the support from others and the listening of family members allowed to individuals with fibromyalgia to have a better experience of lockdown: “*I find that we receive more social support during this period” (Marie, 50 years old)*. The exceptional situation experienced by all French people has enabled those suffering from fibromyalgia to regain their place in society: “*Even some friends we manage to call them, so we think of others better too and what they may go through, we can't just think of ourselves” (François, 58 years old)*. Fibromyalgia stopped being a main priority.

“*The lockdown is positive, it's going well, we live it well” (Gabrielle, 52 years old)*. Finally, it seemed that home lockdown was experienced positively by fibromyalgia patients. This period allowed them to organize their time while respecting their rhythm of life: “*Lockdown doesn't stress me it's even positive […] I rest and I manage my time as I wish” (Bernard, 53 years old)*. The suspension of the usual social constraints promoted the management of their daily life and positively impacted their morale. Being confined on the same basis as the general population allowed them to better contend with the pathology. They were in greater spirits: “*Well, morally, I would even say that it is better than before the lockdown” (Fabienne, 52 years old)*.

## Discussion

The objective of this work was to understand impact of lockdown on the daily life of fibromyalgia patients and their experience with adapted physical activity. In this novel situation of lockdown for the French population, fibromyalgia patients could have suffered from the multiple health consequences that it imposed. Fibromyalgia patients are already subject to high anxiety levels (Castelli et al., 2012), sleep disorders (Osorio et al., 2006), and the first recommended therapeutic is actually the regular practice of physical activity.

Our results clearly showed that fibromyalgia sufferers are faced with a lot of difficulties. They reported an increase in the triad symptoms (pain, fatigue and sleep disorders) and they also evoked an increase in stress, whether economic, familial or even medical. Likewise, a recent study showed an increase in pain in fibromyalgia patients, which was associated with an increased level of anxiety (Kharko et al., 2020). This is consistent with another study in which people with chronic diseases were at greater risk of distress and somatization than “healthy” subjects during the period of lockdown (Louvardi et al., 2020). Thus, the impact of lockdown on fibromyalgia patients is real but is ultimately not specific to this pathology. Patients suffering from non-communicable diseases are concerned by these same difficulties (Palmer et al., 2020), as the general population.

Regarding the practice of physical activity, the closure of clubs, associations and physiotherapy offices forced the cessation of supervised and adapted physical activities. The objective of the Fimouv program is to promote the practice of adapted physical activity among fibromyalgia patients and to make it last over time. If the majority of patients managed to set up physical activities independently, most regrets stopping their sessions and even point out a lack of practice. While this was not necessarily the case before they joined the Fimouv program, now patients link the increase in pain and fatigue to the lack of physical activity, in line with interventions promoting physical activity to reduce these symptoms (Bidonde et al., 2019; Bodéré et al., 2020). It would seem that the forced cessation of physical activity had shed light on the promotion carried out by health professionals of the program by allowing patients to realize the importance and the benefit of physical activity on the symptoms of fibromyalgia.

The autonomous implementation of a physical activity at home was possible thanks to the possibility of adapting the pace of life to fluctuations in fibromyalgia symptoms and to individual circadian rhythms. Lockdown led to a decrease in social rhythms such as transportation and work schedules and times, children's school rhythms, eating rhythms, etc. Thus, the majority of time constraints were reduced, leaving more room for spare time devoted to leisure, social relations, physical activities, as well as to rest. The literature has shown that these imposed rhythms are not always beneficial to health and can even promote suicidal thoughts (Aveline et al., 1984). In our study, the interruption of social rhythms engendered by lockdown seemed profitable for fibromyalgia patients, who took the time to listen to their body, and to establish a rhythm for one's self. Cavalli et al. (2021) reported that the introduction of new daily routines, with more flexible and less stressful work, and requiring less travel, left more room for the practice of regular physical activity and was associated with an improvement in the fibromyalgia symptoms during lockdown (Cavalli et al., 2021). Although there is not yet any data available on this topic, it would seem that the reduction in professional, social and family constraints is beneficial to the management of the pathology and its personal experience.

Finally, the lockdown seemed to have a rather positive impact on patient morale. An explanation could lie in the importance of social bonding and perceived social support. One of the consequences of fibromyalgia is social isolation because patients can no longer keep up with the pace imposed by society, with a strong sense of guilt and withdrawal (Galvez-Sánchez et al., 2019). During lockdown, the whole French population had to respect the rules of isolation: there were no more social differences, familial rhythms matched, and ultimately patients felt more integrated and supported by their loved ones. In a qualitative study on patients with chronic fatigue syndrome, similar results were reported, highlighting both the increase in social interactions thanks to social networks, a less demanding way of communicating with loved ones, and a lessened feeling of guilt for refusing or canceling invitations (Brewer and Stratton, 2020). The positive social experience of our patients allowed them to feel more connected with society and to finally feel “like everyone else.” However, when the end of lockdown was announced, patients expressed a return of stress in anticipation of social pressure and less adapted life rhythms of which they would have to face again. We can compare these results to the “cabin fever syndrome” defined since the 20th century as an apprehension or even fear of leaving home after a long period of isolation, involving feelings of irritability and stress (Rosenblatt et al., 1984).

Throughout this study, we wanted to give an overview of the quality of life of fibromyalgia patients during lockdown. The main strength of our study was that we conducted the interviews over a period of five weeks, in a rapidly changing health situation. Using the inductive method, we regularly went back and forth with the initial interview guide in order to always be in touch with government announcements regarding the evolution of the epidemic. However, the lockdown situation forced us to adapt our investigation methods. Consequently, we had to carry out our interviews in non-standard conditions and all the participants did not benefit from the same interview circumstances. Phone interviews also had the disadvantage of masking all the part of the gestural and non-verbal communication, which is sometimes very revealing. Another limitation was the selection procedure of the sample. We recruited all our interviewees on the Fimouv study, which implies that patients are active in their care and certainly more motivated. But we contacted all our patients, even the ones who dropped-outs. To limit this motivation bias, we chose to contact only patients who completed at least 3 months of the Fimouv study. It was the end of the interventional phasis and we expected autonomy in care.

## Conclusion

Lockdown imposed fewer constraints on the pace of life and was thus better experienced by fibromyalgia patients. Reducing social demands could be a key for fibromyalgia management, by adapting the pace of life (family, professional, social, administrative, etc.). Indeed, when constrained rhythms were reduced or even non-existent, pain and fatigue, which are the primary symptoms of fibromyalgia, could take a back seat thanks to better morale. If this period of lockdown was rather well-lived, the announcement of the end of the lockdown seemed to accentuate the stress and symptoms. Also, it could be interesting to do a post-containment assessment to measure the longer-term impact of this unprecedented period.

We have noticed the importance of the continuity of Fimouv monitoring. Indeed, patients felt listened to and recognized as suffering, despite the invisible nature of the pathology. Telemedicine could therefore constitute a very relevant monitoring tool for fibromyalgia patients, making it possible both to continue the medical follow-up initiated and to reduce travel constraints which can be heavy for some patients. Also, a line of research could be the development of tele-rehabilitation for fibromyalgia patients, which has been tested in other pathologies and has shown physical effects comparable to those of conventional rehabilitation (Chan et al., 2016).

## Data Availability Statement

The original contributions presented in the study are included in the article/Supplementary Material, further inquiries can be directed to the corresponding author/s.

## Ethics Statement

The studies involving human participants were reviewed and approved by IRBN522020/CHUSTE. The patients/participants provided their written informed consent to participate in this study.

## Author Contributions

The Fimouv investigators from University Hospital of Saint-Etienne contributed to the conception and design of the study. AJ and JG contributed to the acquisition of data. CC and AJ wrote the first draft of the manuscript. JG, NB, and DH provided critical revision for intellectual content and oversight. All authors contributed to the article and approved the submitted version.

## Conflict of Interest

The authors declare that the research was conducted in the absence of any commercial or financial relationships that could be construed as a potential conflict of interest.
