# Supplementary material for: Understanding Experiences of Fibromyalgia Patients Involved in the Fimouv Study During COVID-19 Lockdown
Source: Front Psychol. 2021 Jul 20;12:645092. doi: 10.3389/fpsyg.2021.645092 (PMC8329548; doi:10.3389/fpsyg.2021.645092)
Supplement: Supplementary file 1 [file Data_Sheet_1.PDF]

Supplementary Material

1 Semistructured interview guide

THEMES	QUESTIONS	REMINDERS
**Presentation**	1. Can you tell me about yourself, and where you live?	- Age<br>- Living environment (rural, urban)<br>- Type of residence (apartment, house, etc.)<br>- Outside access (balcony, garden, etc.)
**Experience in home-based lockdown**	2. How do you feel in this home-based lockdown period?	Has this lockdown changed anything in your daily life?
	3. How you feel regarding pain, fatigue, etc.?	Are you suffering from sleep disorders? Is your morale impacted?
	4. Has your pathology management been modified during the lockdown?	How has it been change? Is the medical monitoring more difficult? Is treatment more difficult to obtain? Were the health professionals requisitioned?
	5. Did the lockdown modify your daily life?	Have you developed new habits? New hygiene behavior? New purchases? Restrictions in consumption or trips?
**COVID-19**	6. Have you been infected by this virus?	If yes, how was your care? Have you been hospitalized?
	7. Have your loved ones been infected by this virus?	If yes, are you in direct contact with them?
	8. Are you afraid to contract this virus? Are you worried for your family?	Do you feel vulnerable regarding this virus?
**Social support and environment**	9. In which conditions are you spending your lockdown?	Is the number of people present in the household usual?<br>Have your children returned to the family home during the lockdown?
	10. How is daily life at home during this period of lockdown?	Is it conducive to more rest?<br>More moments of family sharing? Tensions? Disputes?
	11. Are you working currently?	If so, have your working conditions been changed? Are you teleworking?<br>If not, for what reasons? Is it related to lockdown or to a work stoppage due to the pathology?
	12. Would you say that your social relations have evolved since the lockdown?	How have they changed? How do you communicate (video call, phone, messaging)?<br>Do you feel that you are receiving more/less support?

	13. Have you felt more stressed since the start of lockdown?	If so, why?
	14. Do you have the feeling that your entourage is there for you?	Is your entourage worried about you? Are they considerate? Are they listening?
**Adapted physical activity**	15. You participated in the Fimouv study at the CHU of Saint-Etienne. Which group were you in?	When were you included in the program?<br>What did you do in this program?<br>Did you attend all of the sessions?
	16. Did this study / program encourage you to engage in regular physical activity?	
	17. Have you practiced physical activity during this period of lockdown?	Have your sports habits changed during this period of lockdown? If yes, how?<br>Have you put alternatives in place to continue exercising? Did your sports club give you solutions to practice during this period (follow up, video sessions, etc.)?
	18. Do your loved ones encourage you to participate in physical activity?	If so, do you practice together?
**Post-lockdown**	19. How do you consider the post-lockdown?	Do you think you are changing some of your behaviors?<br>Do you think you are maintaining certain behaviors that you have put in place?
	20. Do you think that it is necessary to adopt more preventive behaviors to avoid this type of event to happen again?	Do you think we should wear masks in medical settings (waiting room, hospital, etc.)? Do you think we should wear masks on public transportation? Do you think that hydro-alcoholic gel should be made available in public places (supermarkets, restaurants, shops, public transportation, etc.)? Do you think we should disinfect our groceries every time?
	21. Do you think that home lockdown had other impacts than those related to COVID-19?	If so, which ones? On our environment? In what way(s)?
**Conclusion**	22. Would you like to add something or bring up a subject that we have not spoken about?	
